# CAP1/CAP2 at the Actin Filament Barbed End: Reconciling Kinetic, Single-Molecule, Structural and Cellular Evidence

**DOI:** 10.3390/cells15201858

**Published:** 2026-10-13

**Authors:** Thanh Huu Phan Ngo, Wan Lee

**Affiliations:** 1 Department of Biochemistry, Dongguk University College of Medicine, 123 Dongdae-ro, Gyeongju 38066, Republic of Korea; thngo139@gmail.com; 2 Section of Molecular and Cellular Medicine, Medical Institute of Dongguk University, Dongguk University College of Medicine, 123 Dongdae-ro, Gyeongju 38066, Republic of Korea

**Keywords:** cyclase-associated protein, actin dynamics, barbed end, depolymerization, single-molecule imaging

## Abstract

**Highlights:**

**What are the main conclusions?**

- C-CAP accelerates actin barbed-end depolymerization, with strong support for WH2 involvement. A direct CARP contribution remains unresolved because published constructs also differ in scaffold and valency.
- Reported effects of CAP on formin behavior remain context-dependent. Processive barbed-end behavior is supported for yeast Srv2ΔCARP, whereas mammalian CAP requires direct residence measurements.

**What are the implications of the main conclusions?**

- Structural and high-speed atomic force microscopy studies constrain current models but do not independently resolve how C-CAP engages the barbed end.
- Monomer availability, filament nucleotide state, barbed-end occupancy, and CAP abundance motivate prospective cellular tests; tropomyosin decoration is an additional hypothesis.

**Abstract:**

Cyclase-associated proteins CAP1 and CAP2 regulate actin monomer recycling and cooperate with cofilin at filament pointed ends. Recent reconstitution studies show that the C-terminal region also accelerates barbed-end depolymerization, but the underlying mechanism remains unsettled. A central unresolved issue is whether the CARP domain contributes directly. Published constructs change CARP content together with scaffold architecture or valency, so they do not isolate this variable. We therefore specify matched construct series that would test CARP contribution directly. We also evaluate conflicting formin results and the evidence for processivity, with explicit attention to labeling, surface effects, filament state, accessory proteins, and species or construct differences. The evidence strongly supports C-CAP activity and WH2 involvement, whereas CARP contribution, the basis of the formin discrepancy, and mammalian CAP processivity require further testing. Structural and high-speed atomic force microscopy studies narrow the possible mechanisms but do not independently establish C-CAP engagement at the barbed end, and cellular studies have not directly measured C-CAP occupancy or activity at barbed ends. Monomer availability, filament nucleotide state, barbed-end occupancy, and local CAP abundance are therefore treated as candidate variables for prospective cellular tests. Tropomyosin decoration is an additional hypothesis rather than an established determinant.

## 1. Introduction

Actin filaments are polar polymers whose barbed and pointed ends support distinct assembly and disassembly reactions. The barbed end is usually the kinetically favored site for subunit addition, whereas the pointed end exchanges subunits more slowly under many conditions. This polarity underlies classical treadmilling, in which ATP-actin is added mainly at the barbed end and hydrolyzes and releases phosphate within the filament, and older ADP-actin is lost preferentially from the pointed end. Recent work has shown that both ends support more diverse reactions than this classical scheme implies [1,2,3]. Twinfilin, for example, promotes barbed-end depolymerization and capping-protein release [4,5,6], whereas the bacterial effectors VopF and VopL can drive pointed-end elongation [7]. Pointed-end dynamics also contribute to the maintenance of sarcomeric thin filaments [8,9].

Cyclase-associated protein (CAP) is a conserved actin regulator with roles in filament-end dynamics and monomer recycling. Mammals express two paralogs. CAP1 is broadly expressed, whereas CAP2 is enriched in tissues including muscle and brain [10,11]. Both paralogs contain an N-terminal oligomerization domain and helical-folded domain (HFD) and a C-terminal actin-binding region [12,13]. N-CAP cooperates with cofilin at filament pointed ends [14,15]. C-CAP binds ADP-actin [16,17], catalyzes nucleotide exchange [18,19], and supports the transfer of recharged actin to profilin [20]. These activities connect filament disassembly with the regeneration of polymerization-competent ATP-actin.

More recently, a direct role for CAP at filament barbed ends was reported. Alimov and colleagues showed that the C-terminal region of CAP accelerates barbed-end depolymerization and alters formin behavior in single-filament assays [21]. Towsif and Shekhar subsequently confirmed accelerated barbed-end depolymerization using three-color single-molecule imaging and reported effects on capping protein and formin [22]. The studies agree on several central observations but differ on three mechanistically important issues. They assign different roles to the C-terminal CARP domain, report opposite effects on formin processivity, and interpret CAP residence at the barbed end differently.

Two additional studies provide complementary information but do not directly resolve these disagreements. High-speed atomic force microscopy visualized transient interactions of Xenopus CAP1 with actin filaments and showed that actin-monomer loading changes both residence time and binding preference [23]. Cryo-electron microscopy subsequently resolved the structural mechanism of CAP and cofilin at the pointed end and the structural basis of cofilin-mediated capping-protein release [24]. However, those structures did not resolve the C-terminal region of CAP at barbed ends.

These experiments differ in species, protein constructs, filament age, accessory proteins, labeling strategies, and readouts. Mechanistic comparison therefore requires direct observations to be separated from condition-specific interpretations and technical limitations. The key questions are which conclusions remain secure and which variables were actually tested independently.

## 2. CAP Architecture and Established Actin Functions

### 2.1. Domain Organization and Monomer Recycling

CAP is a multidomain protein of about 57 kDa conserved across animals, fungi, and plants [10,12]. Its N-terminal region contains an oligomerization domain and a helical-folded domain (HFD). The C-terminal region contains two proline-rich motifs flanking a WASP-homology 2 (WH2) domain and terminates in the CARP domain [25,26]. CAP1 is broadly expressed, whereas CAP2 is enriched in muscle, neurons, and skin [11]. CAP1 loss is embryonically lethal in mice, and CAP2 deficiency disrupts striated muscle organization and function [27,28,29].

The C-terminal region regulates actin monomers. The WH2 domain binds ATP- and ADP-actin with similar micromolar affinity [18]. The CARP domain strongly prefers ADP-actin and contributes to nucleotide exchange [16,17]. Structural and biochemical studies show that the C-terminal region accepts ADP-actin released during filament turnover, promotes nucleotide exchange, and facilitates transfer of ATP-actin to profilin [17,20]. The CARP domain alone can promote nucleotide exchange, whereas the WH2 domain becomes important when actin is associated with cofilin [13,30].

**Figure 1:**
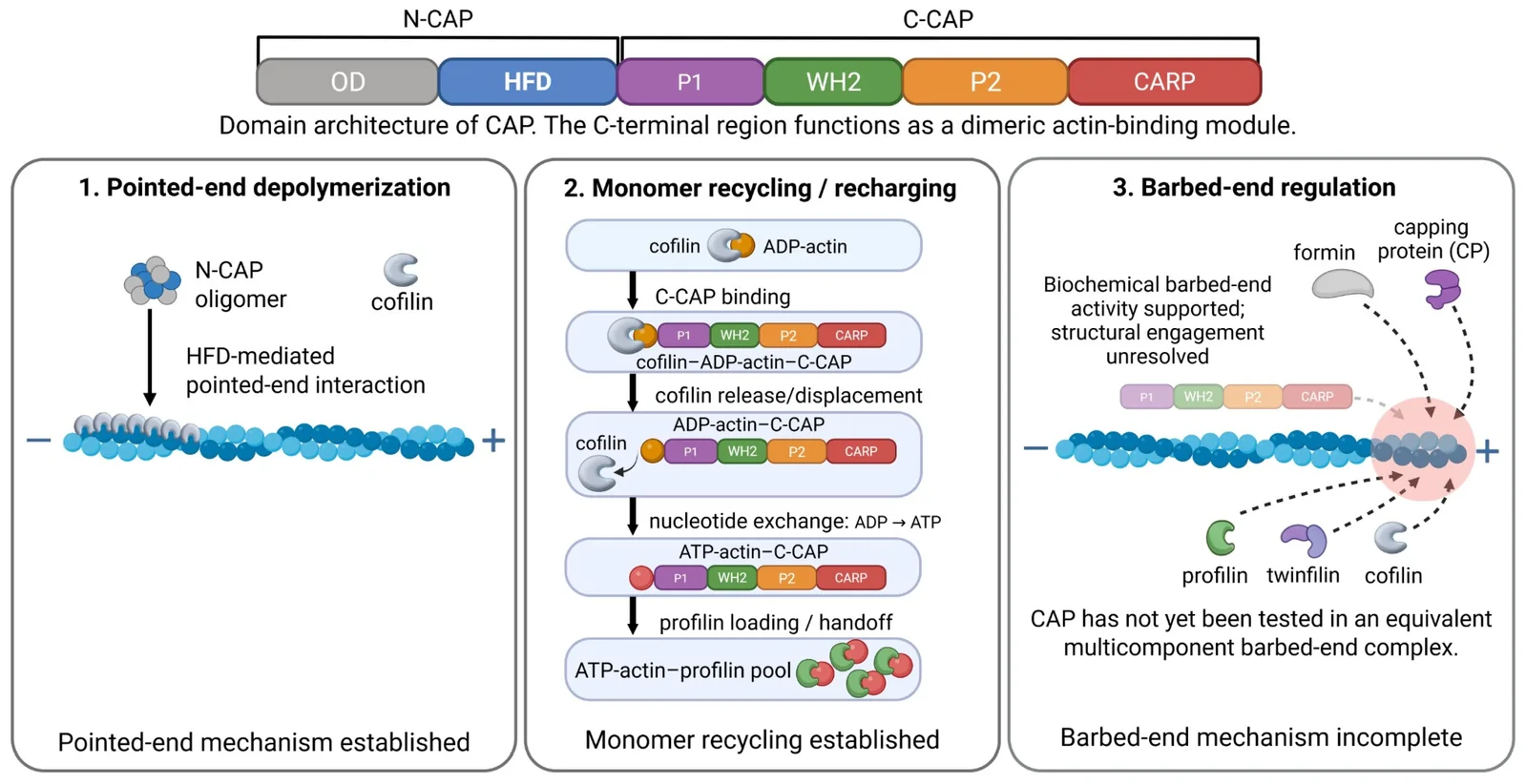
CAP domain architecture and major actin-regulatory functions. (**1**) N-CAP, comprising the oligomerization domain and HFD, oligomerizes and cooperates with cofilin-decorated F-actin to accelerate pointed-end depolymerization; cofilin is shown along the filament. (**2**) C-CAP, comprising P1-WH2-P2-CARP, mediates actin-monomer recycling, including cofilin release, nucleotide exchange, and transfer of ATP-actin to profilin; orange and red spherical actin icons denote ADP-actin and ATP-actin, respectively. (**3**) C-CAP has barbed-end activity in reconstituted systems, but its structural engagement and the direct contribution of CARP remain unresolved. Dashed relationships indicate contextual or untested interactions. The N-CAP oligomer is schematic and does not imply a fixed stoichiometry. Solid arrows indicate established interactions or sequential steps, whereas dashed arrows indicate contextual or untested relationships. Created in BioRender. Lee, W. (2026) https://BioRender.com/iy5c8ga.

This activity links filament disassembly to the intracellular monomer pool. In reconstituted motility systems, sustained actin turnover fails without CAP-dependent monomer recycling [31,32]. CAP can therefore influence actin dynamics through two conceptually distinct routes. It can act directly on filament ends, and it can alter the supply and nucleotide state of soluble actin available for subsequent assembly [33]. Figure 1 summarizes CAP domain organization and these established functions, together with the more recently described barbed-end activity.

### 2.2. Pointed-End Depolymerization and Oligomerization

The N-terminal HFD mediates the best-established filament-end activity of CAP. CAP alone modestly accelerates pointed-end depolymerization, but with cofilin the rate increases by more than two orders of magnitude [14,15]. Cryo-electron microscopy has provided a structural explanation for this synergy [24].

At the pointed end, binding of one HFD produces little structural disruption. Binding of a second HFD to the adjacent terminal subunit creates steric strain and shifts the terminal actin subunit. Cofilin increases this effect by changing the filament twist. In the combined complex, the terminal subunit is displaced further and can leave together with bound cofilin and HFD [24]. The structural observations are therefore consistent with the large kinetic synergy measured in independent single-filament experiments [14,15].

Oligomerization likely facilitates this multivalent interaction, but the exact higher-order state of CAP remains unsettled. Yeast Srv2 has been reported as a five- to six-subunit complex and has been visualized as a sixfold assembly [34,35]. Several measurements of mammalian CAP favor a tetramer, including the native mass spectrometry of N-terminal fragments and mass photometry of full-length human CAP in an actin-containing complex [24,36]. Other mammalian reconstitution studies have retained a hexameric model [13,37]. Structural work on the isolated N-terminal domain also supports variable oligomerization [38]. No single study has compared yeast and mammalian full-length proteins under identical conditions.

For this reason, higher-order N-CAP organization is best treated schematically rather than assigned a universal stoichiometry. More importantly, oligomerization should not be assumed to have the same functional requirement in every CAP reaction. Defined-valency experiments show that increasing oligomerization can enhance actin-regulatory activity, but the effect depends on the assay [34,35,36]. This point becomes important when evaluating C-terminal constructs used to test CAP activity at barbed ends.

## 3. CAP at the Actin Filament Barbed End

**Figure 2:**
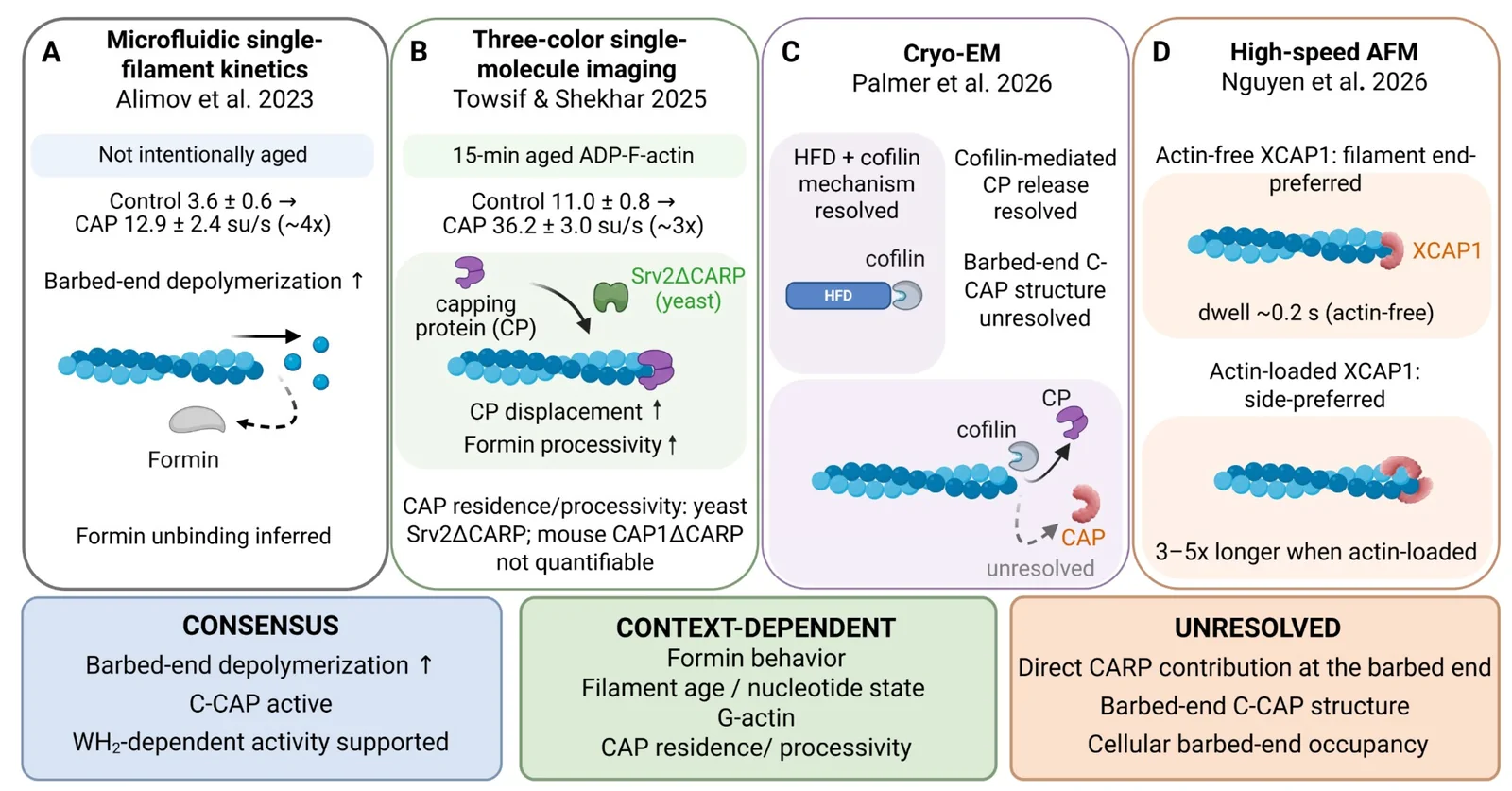
Experimental approaches used to examine CAP at actin filament ends. (**A**) Alimov et al. [21] used microfluidic single-filament kinetics; formin occupancy was inferred from filament behavior, and formin unbinding was concluded without directly labeling formin. (**B**) Towsif and Shekhar [22] used three-color single-molecule imaging with fluorescently labeled formin and measured increased formin processivity and capping-protein displacement. CAP residence/processivity was quantified with yeast Srv2ΔCARP because labeled mouse CAP1ΔCARP could not be quantified reliably. (**C**) Cryo-electron microscopy [24] resolves the pointed-end HFD/cofilin mechanism and cofilin-mediated capping-protein release but does not resolve C-CAP at the barbed end. (**D**) High-speed AFM [23] shows a moderate end preference for actin-free XCAP1 (Xenopus laevis CAP1; dwell time approximately 0.2 s) and side-preferred binding after actin loading, with a 3- to 5-fold longer dwell time. Surface attachment and scanning force are method-specific limitations. The lower boxes separate points of consensus from context-dependent conclusions and unresolved questions. Solid arrows indicate direct observations, whereas dashed arrows indicate interpretation or unresolved mechanism. Created in BioRender. Lee, W. (2026) https://BioRender.com/3wlvo4r.

Four recent studies provide complementary views of CAP at actin filament ends. Two directly measure barbed-end depolymerization [21,22], one visualizes CAP–filament interactions by high-speed atomic force microscopy [23], and one resolves structural mechanisms at filament ends by cryo-electron microscopy [24]. Figure 2 summarizes what each approach directly establishes and where important uncertainties remain.

### 3.1. What the Reconstitution Studies Establish

Two studies directly examined CAP-dependent barbed-end depolymerization using single-filament approaches [21,22]. Despite differences in experimental design, they agree on two central points. CAP accelerates barbed-end depolymerization, and the activity resides in the C-terminal region with strong dependence on the WH2 domain.

The studies reported different absolute depolymerization rates. Alimov and colleagues used filaments that were not deliberately aged and measured a control rate of 3.58 ± 0.59 subunits per second [21]. Towsif and Shekhar aged filaments for 15 min to enrich ADP-F-actin and measured 11.0 ± 0.8 subunits per second [22]. CAP increased these rates to 12.9 ± 2.42 and 36.2 ± 3.0 subunits per second, respectively. The reported fold accelerations were comparable despite different baseline rates. Filament age is linked to nucleotide state because phosphate release converts newly assembled ADP-Pi filaments to older ADP-F-actin. Both studies also examined ADP-Pi or phosphate-buffered conditions [21,22], so nucleotide state is relevant to depolymerization kinetics but does not resolve the opposite formin conclusions. The protocols differed in more than aging, and no matched experiment has shown that filament age alone accounts for the baseline difference.

Both studies used microfluidics-assisted single-filament imaging but differed in how formin occupancy was monitored. Alimov and colleagues inferred formin occupancy from filament growth or fluorescence without labeling formin [21]. Towsif and Shekhar labeled formin and visualized its presence at the barbed end [22]. Neither strategy is intrinsically superior. The unlabeled approach avoids a fluorophore on formin, whereas labeling provides an independent occupancy signal but may itself alter formin behavior. Differences in filament state, construct design, and surface configuration also prevent a one-variable comparison.

Together, the studies establish that C-CAP can accelerate barbed-end depolymerization and that WH2 is a major determinant of this activity. Their technical differences do not by themselves resolve the formin disagreement. Table 1 summarizes the principal design differences, direct readouts, and technical limitations.

### 3.2. Does the CARP Domain Contribute Directly?

The strongest unresolved domain question concerns CARP. Alimov and colleagues concluded that both WH2 and CARP contribute to C-CAP-mediated barbed-end depolymerization [21], whereas Towsif and Shekhar found that CAP lacking CARP retained substantial barbed-end depolymerization activity [22]. However, the experiments did not test CARP as the only variable.

Alimov and colleagues first showed that a WH2 point mutant that disrupts actin binding abolishes barbed-end depolymerization. This provides direct evidence that WH2 is necessary for the activity measured in that assay. To test CARP, they used a P1-WH2-P2 fragment that lacked CARP and was artificially dimerized with GST. The fragment did not reproduce full C-CAP barbed-end depolymerization. This result is consistent with a CARP contribution, but it does not establish that CARP itself is required because GST also imposes a non-native scaffold and geometry.

Towsif and Shekhar used CAPΔCARP, which retains the N-terminal oligomerization machinery and all domains except CARP [22]. The construct retained substantial barbed-end activity. This argues against an absolute CARP requirement under those conditions. However, the construct also differs substantially from the GST-dimerized fragment used in the earlier study. Its oligomeric state and geometry did not match the earlier construct.

CAP activity can depend on valency, although the relationship is assay-dependent. In the defined-valency pointed-end assay of Kotila et al., monomeric HFD required much higher concentrations for efficient cofilin-dependent pointed-end depolymerization, whereas GST-mediated dimerization markedly increased activity, and oligomeric N-CAP or full-length CAP was effective at lower concentrations [14]. Studies of Srv2 and mammalian CAP likewise show that oligomerization can alter activity without establishing a universal valency threshold [34,35,36]. Valency is therefore a credible confounder in the barbed-end comparison, but it does not by itself explain the different CARP conclusions.

**Figure 3:**
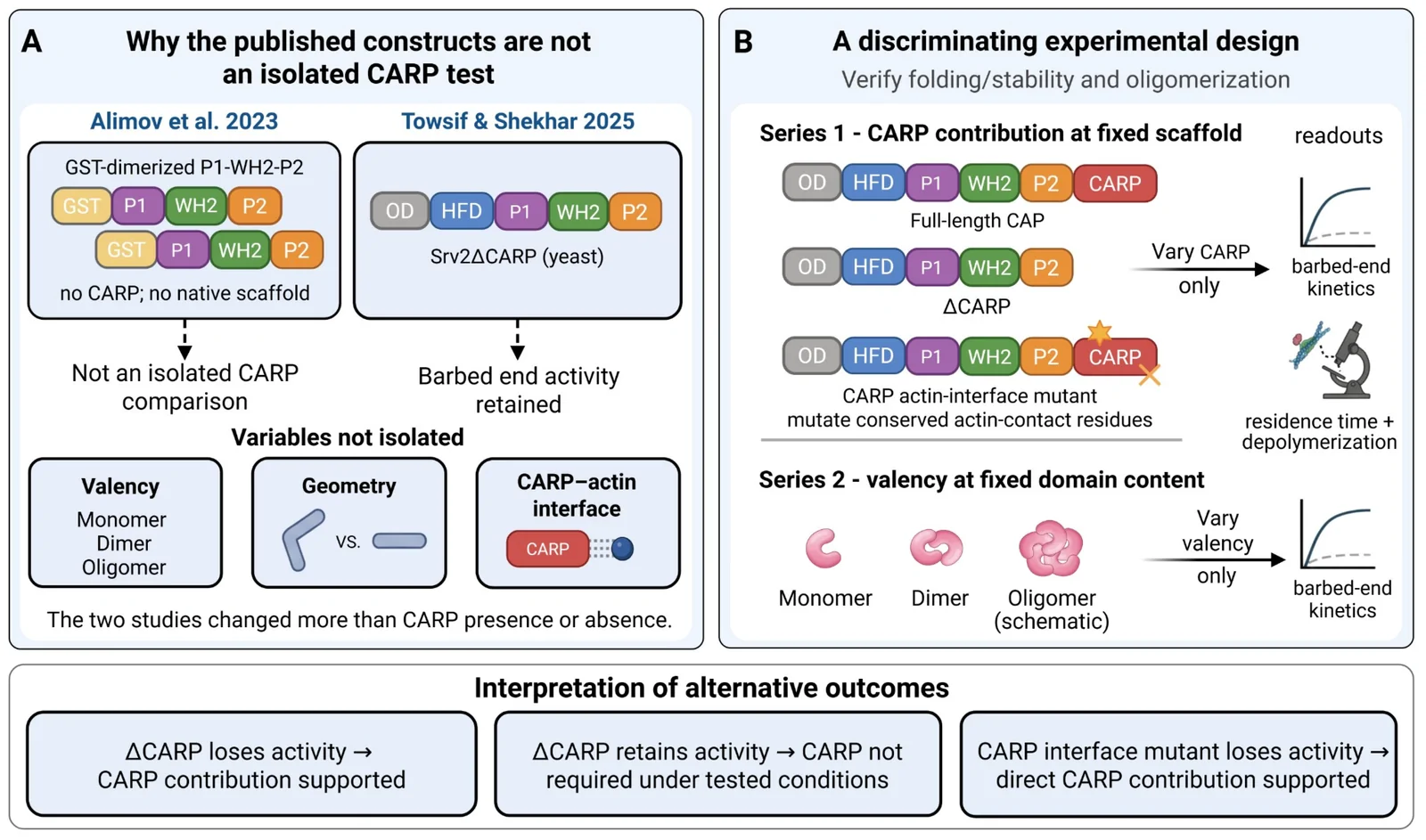
Experimental design to separate CARP-specific effects from scaffold and valency. (**A**) The GST-dimerized P1-WH2-P2 fragment [21] and Srv2ΔCARP [22] differ in CARP content, architecture, and oligomerization context, so the published comparison does not isolate CARP. Valency, geometry, and the CARP–actin interface are therefore shown as variables that were not independently controlled. (**B**) A matched series comparing full-length CAP, ΔCARP, and a CARP–actin interface mutant would test CARP contribution at a fixed scaffold. A second scaffold-matched series would compare monomeric, dimeric, and oligomeric P1-WH2-P2 at fixed domain content. The pink monomer, dimer, and oligomer symbols are schematic valency icons; the oligomer does not imply a fixed stoichiometry or structural arrangement. Barbed-end kinetics, direct residence measurements, and depolymerization should be measured under matched buffer and filament-aging conditions, with protein stability and oligomerization verified. In panel A, dashed arrows connect the published constructs to their interpretive consequences. In panel B, solid arrows indicate the proposed controlled comparisons and readouts. Alternative outcomes and their interpretations are shown at the bottom. Created in BioRender. Lee, W. (2026) https://BioRender.com/xc9073w.

A decisive experiment should separate CARP contribution from scaffold and valency. First, compare full-length CAP, ΔCARP, and a CARP–actin interface mutant in the same scaffold, while verifying protein stability and oligomerization. Conserved CARP residues known to disrupt actin binding provide a starting point for the interface mutant [30]. In parallel, scaffold-matched P1-WH2-P2 monomeric, dimeric, and oligomeric constructs should be compared so that valency is varied without changing domain content. These experiments would distinguish a CARP-specific contribution from effects caused by oligomerization or construct geometry. Figure 3 summarizes this matched design and the alternative outcomes.

### 3.3. Formin and Capping Protein

The two reconstitution studies report opposite conclusions about CAP and formin. Alimov and colleagues concluded that CAP promotes formin unbinding [21], whereas Towsif and Shekhar observed an approximately tenfold increase in formin processivity [22].

The discrepancy is not explained by the presence or absence of profilin-actin. Towsif and Shekhar reproduced the increase in formin processivity under elongation-promoting conditions containing profilin-actin (their SI Appendix, Figure S3) [22]. The assays nevertheless differ in how formin occupancy was monitored. Alimov and colleagues inferred occupancy from filament growth or fluorescence without labeling formin [21], whereas Towsif and Shekhar followed fluorescently labeled formin [22]. The first approach avoids a fluorophore on formin but infers occupancy from filament behavior; the second provides a direct formin signal but introduces a label that may alter processivity or elongation. The assays also sampled different barbed-end reaction states, including elongating and depolymerizing conditions. The current data therefore establish a genuine formin discrepancy that is not resolved by profilin-actin conditions or by assuming that one tracking method is superior.

CAP can also influence profilin and actin monomers, but these interactions should not be used to explain the opposite formin conclusions because the Towsif result persists in the presence of profilin-actin [22].

Capping protein has been studied more directly in later work. C-CAP accelerated capping-protein dissociation, and the effect was attributed largely to WH2 [22]. Cryo-electron microscopy independently showed that cofilin changes terminal-actin conformation and weakens capping-protein binding [24]. This cofilin-mediated route is distinct from C-CAP-mediated uncapping and does not establish the mechanism of CAP-dependent CP release. Earlier studies on CAP/acetylated-actin regulation of INF2 [39,40], together with later evidence for monomer-buffering effects on INF2 [41], concern a different regulatory system from the mDia constructs lacking the DID autoinhibitory domain used in the two barbed-end studies; these INF2 results therefore do not adjudicate the mDia formin discrepancy.

### 3.4. Processivity and the Need for a Mammalian CAP Measurement

Towsif and Shekhar proposed that Srv2/CAP acts as a processive barbed-end depolymerase [22]. For Cy5-Srv2ΔCARP, a mean residence time of 226 ± 54 s and a bound-end depolymerization rate of 22.9 ± 4.6 subunits per second yielded an estimated 5180 ± 1620 subunits removed per binding event. Estimating run length from residence time and depolymerization rate is appropriate for these data and supports processive behavior of yeast Srv2ΔCARP.

The key limitation is species and construct identity. The long residence time was measured with yeast Srv2ΔCARP because fluorescent mouse CAP1ΔCARP showed nonspecific coverslip binding and could not be quantified reliably [22]. Thus, the study supports processivity for yeast Srv2ΔCARP but does not yet establish whether mammalian CAP has a comparable residence time or run length.

A direct measurement of fluorescent mammalian CAP at barbed ends remains valuable, with explicit photobleaching controls and reporting of censored binding events. These controls would test whether the long residence is reproduced with mammalian CAP without challenging the calculation used to estimate processivity.

### 3.5. What Structural and AFM Studies Add

The 2026 cryo-electron microscopy study provides a detailed mechanism for CAP and cofilin at the pointed end but does not resolve the C-terminal region of CAP at the barbed end [24]. Of the twelve reported structures, the barbed-end maps contain cofilin and/or capping protein but no resolved CAP density. Full-length CAP was included in an initial preparation, yet only the HFD was resolved. The structural data therefore answer a different question from the C-CAP reconstitution studies. They resolve the pointed-end HFD mechanism and cofilin-mediated uncapping but do not provide a structural test of C-CAP binding at the barbed end.

This result should not be interpreted as evidence that C-CAP does not bind the barbed end. The structural study lacked a resolved C-CAP density at that site and therefore does not distinguish between absence, low occupancy, conformational heterogeneity, and insufficient resolution.

The same structures provide strong evidence for a distinct cofilin-mediated route to capping-protein release. Cofilin alters the conformation and twist of terminal actin subunits and progressively weakens capping-protein interactions [24]. This mechanism should not be treated as evidence for the mechanism of C-CAP-dependent uncapping [22].

High-speed atomic force microscopy provides another type of evidence [23]. Actin-free Xenopus CAP1 showed a modest preference for filament ends, whereas actin-loaded CAP1 showed longer residence and predominant filament-side binding. By contrast, the side binding of fluorescent CAP/Srv2 was not reported in the single-molecule TIRF study [22]. The methods probe different preparations and have different artifacts. High-speed AFM requires surface attachment and repeated scanning by the AFM tip, so adsorption geometry and imaging force can perturb weak or transient interactions; TIRF has its own surface and fluorescent-labeling constraints. The apparent difference in side binding therefore remains unresolved and should be tested under more closely matched conditions.

### 3.6. CAP and Twinfilin at a Shared Regulatory Site

Twinfilin and CAP have overlapping activities at filament ends. Twinfilin can accelerate barbed-end depolymerization and promote capping-protein release [4,5,6]. C-CAP also accelerates barbed-end depolymerization and capping-protein dissociation [21,22]. Their direct relationship remains uncertain.

Earlier work showed strong cooperation between yeast Twinfilin and yeast Srv2 at both filament ends, with especially rapid pointed-end depolymerization [37]. The mammalian relationship is more restricted. Mouse N-CAP1 did not generally accelerate mouse Twinfilin at both ends, although at the barbed end a low concentration of mTwf1 that was inactive alone became effective when combined with N-CAP1 [37]. Twinfilin can also depolymerize barbed ends without CAP [5,42]. These results support condition- and species-dependent cooperation rather than an obligatory CAP–twinfilin complex.

Cooperation, independence, and competition therefore remain possible in different reaction states. Multicomponent imaging has shown that formin, capping protein, and twinfilin can transiently occupy one barbed end and that the effect of twinfilin depends strongly on the accompanying proteins [43]. CAP has not been tested in an equivalent multicomponent configuration. Direct reconstitution with CAP, twinfilin, and capping protein, with or without formin, would distinguish these alternatives more effectively than additional pairwise assays.

## 4. From Reconstitution to Cells

**Figure 4:**
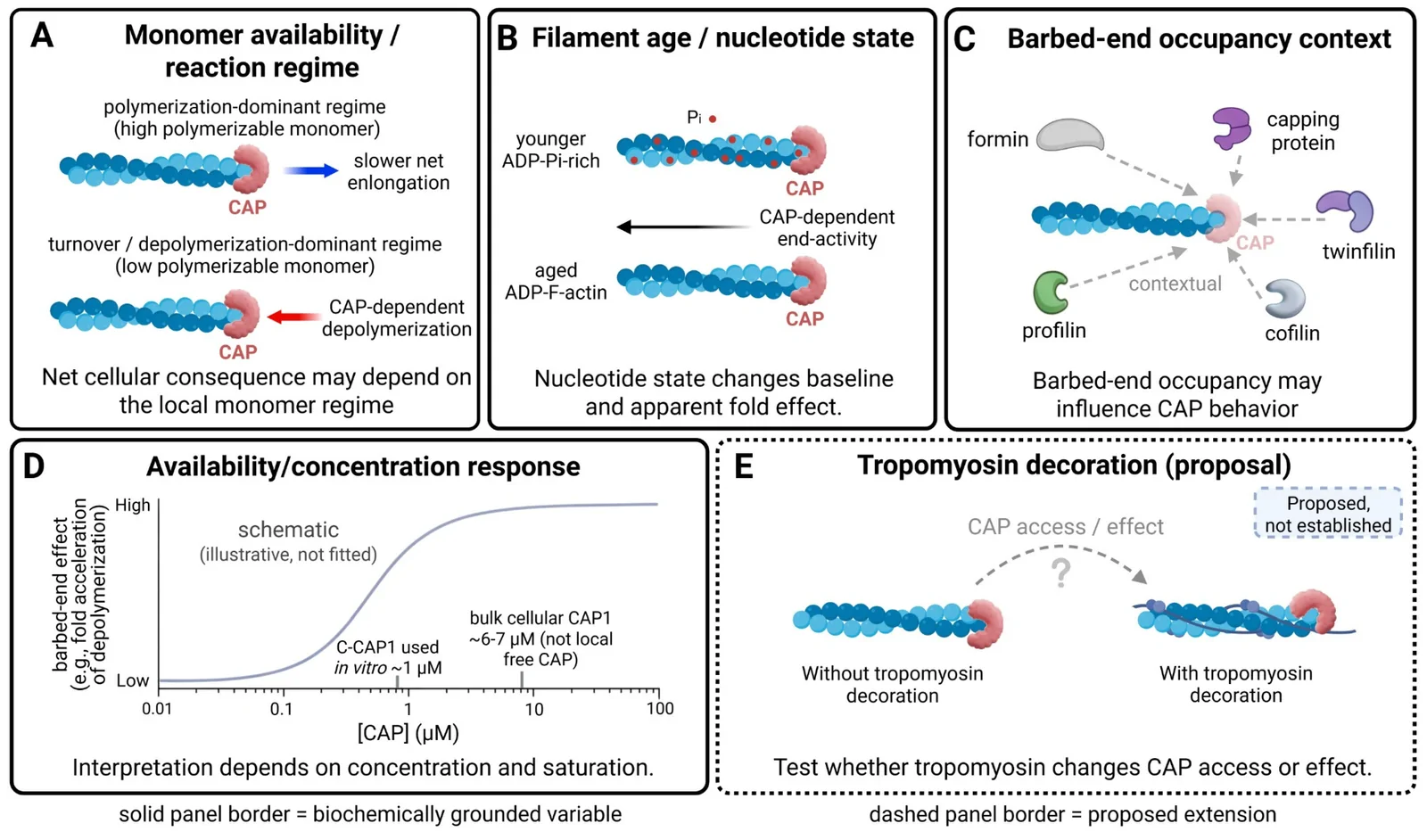
Experimentally motivated variables for prospective tests of CAP function in cells. (**A**) Monomer availability can shift the net reaction regime: high polymerizable-monomer conditions can yield slower net elongation, whereas low polymerizable-monomer conditions can favor CAP-dependent depolymerization. (**B**) Filament age is linked to nucleotide state, illustrated by younger ADP-Pi-rich and aged ADP-F-actin filaments, and can change baseline depolymerization kinetics and the apparent fold effect of CAP. (**C**) Barbed-end occupancy by formin, capping protein, profilin, twinfilin, or cofilin may influence CAP behavior. (**D**) CAP concentration can alter the magnitude and saturation of the response; the plotted curve is schematic rather than fitted, and bulk cellular CAP1 abundance is not equivalent to local free CAP. (**E**) Tropomyosin decoration is shown separately as a proposed, not established, extension. Solid panel borders denote biochemically grounded variables, whereas the dashed border denotes the proposed extension. Panels (**A**–**D**) depict experimentally motivated variables rather than established in vivo determinants. In panel A, the blue and red solid arrows denote slower net elongation and CAP-dependent depolymerization, respectively. Gray dashed arrows in panels C and E indicate contextual or proposed relationships. Created in BioRender. Lee, W. (2026) https://BioRender.com/b4azh36.

The biochemical variables described above are established modifiers of CAP-dependent reactions in reconstituted systems, but they have not been validated as determinants of CAP behavior in intact cells. They are therefore treated here as candidate variables for prospective testing rather than as requirements for interpreting existing cellular phenotypes. Monomer availability, filament nucleotide state, barbed-end occupancy, and local CAP concentration are the primary candidates; tropomyosin decoration is considered separately as a proposed extension. Figure 4 summarizes these candidate variables without assigning them as established cellular determinants.

### 4.1. Monomer Availability and Filament Nucleotide State

CAP can influence actin dynamics both by acting at filament ends and by regulating the soluble actin pool. These two functions make monomer availability an important variable in reconstituted systems. C-CAP can slow net polymerization or promote net depolymerization depending on the concentration of polymerization-competent actin [21]. High-speed atomic force microscopy further suggests that actin loading changes CAP binding preference [23]. Actin-free CAP preferentially associated with filament ends, whereas actin-loaded CAP showed greater side binding. These findings establish biochemical sensitivity to the monomer regime, but they do not show that the same transitions determine the direction of a CAP phenotype in cells. A useful cellular test would therefore measure the soluble actin pool and the CAP phenotype in the same preparation rather than infer monomer availability from total F-actin alone.

Filament nucleotide state also changes CAP activity. ADP-Pi filaments depolymerize more slowly than aged ADP filaments, although the relative acceleration produced by CAP can be larger on the younger filament state [21,22,44]. In the later barbed-end study, maximal rates differed by roughly an order of magnitude between ADP-Pi and ADP filaments, while the fold effect of CAP was larger on ADP-Pi [22]. The earlier study showed the same qualitative separation between absolute rate and relative acceleration [21]. This agreement makes nucleotide state one of the better supported variables in vitro. Its cellular importance remains difficult to infer because newly assembled and aged actin populations coexist within the same cell and are not routinely distinguished in CAP perturbation studies.

Cellular relevance should be tested by measuring local soluble actin and filament age together with the response to CAP perturbation. A prospective association between these variables and the direction of the CAP phenotype would support their mechanistic relevance; failure of such an association would indicate that additional determinants are required.

### 4.2. Barbed-End Occupancy

The barbed end is shared by multiple regulators, including formin, capping protein, twinfilin, profilin, and cofilin [43,45,46]. These proteins do not act as independent binary switches. Three-color single-molecule imaging has shown formin, capping protein, and twinfilin simultaneously associated with one barbed end in a short-lived complex [43]. Twinfilin then promotes capping-protein release, allowing formin-dependent elongation to continue. The efficiency of uncapping also depends on the accompanying proteins. Approximately one twinfilin encounter can be sufficient in the formin-containing complex, whereas many more encounters are required at a capped end without formin [43]. This establishes that barbed-end composition can change the apparent function of an individual regulator.

This makes occupancy a plausible candidate variable, but CAP has not yet been placed experimentally into an equivalent multiprotein barbed-end complex. Direct measurements of CAP occupancy together with formin, capping protein, or twinfilin are lacking, and cellular studies rarely quantify these regulators during CAP perturbation. The occupancy model therefore extrapolates from well-established competition among other barbed-end proteins and also considers newer biochemical evidence for CAP.

A discriminating prediction is that the effect of CAP depletion should depend on barbed-end composition within the same cell type. This can be tested by measuring capping protein and twinfilin, along with CAP, under a common imaging or biochemical protocol. If opposite CAP phenotypes persist despite comparable end occupancy, occupancy alone would be insufficient.

### 4.3. CAP Abundance and Filament Decoration

CAP concentration strongly affects its activity in vitro. In the study by Alimov and colleagues, C-CAP1 accelerated ADP-filament depolymerization with an apparent Kapp of approximately 0.85 µM, and 1 µM produced about 75–80% of the maximal effect [21]. A bulk CAP1 concentration of approximately 6–7 µM has been reported in NIH3T3 cells [47]. The comparison shows that commonly used reconstitution concentrations can fall within the responsive range, but it does not establish how much cellular CAP is available to a given filament end. Localization, paralog composition, binding partners, monomer loading, and sequestration can all reduce or redistribute the effective pool. Cellular abundance should therefore be measured locally when possible rather than inferred from total expression alone.

Consequently, fold changes in CAP expression cannot be translated directly into proportional changes in barbed-end activity. Quantitative cellular measurements would be required to determine whether a given CAP pool lies within the steep or saturated part of the relevant response.

Tropomyosin provides a second candidate variable. Its effects on CAP-associated reactions differ among experimental systems [9,37]. Twinfilin together with N-CAP can depolymerize tropomyosin-decorated filaments in yeast and mammalian reconstitutions, while the same decoration protects strongly against cofilin-mediated severing [37]. In cardiac muscle, tropomyosin-stabilized filaments recruit tropomodulin but not CAP2, and CAP2-dependent depolymerization is reduced in a reaction containing cofilin-2 [9]. Because these experiments differ in CAP paralog, accessory proteins, filament organization, and tropomyosin isoform, direct comparison requires matched conditions. A focused experiment would compare full-length CAP1 and CAP2 with defined tropomyosin isoforms under otherwise matched cofilin and twinfilin conditions.

## 5. Cellular Evidence and Prospective Tests

The cellular studies reviewed below establish context-dependent consequences of CAP perturbation, but they do not directly test C-CAP occupancy or activity at barbed ends. They are included here to define the cellular measurements needed to test whether the reconstituted mechanism operates in vivo. The most informative systems are those in which actin state and transcriptional output were measured in the same preparation.

### 5.1. CAP2 Loss Produces Different Actin Responses Across Tissues

CAP2 deficiency produces different effects on the actin monomer pool in different cellular contexts. Mouse embryonic fibroblasts provide the clearest quantitative example [48]. Fluorescence recovery after photobleaching was faster in CAP2-null cells, with a mean half-recovery time of 8.95 s compared with 14.73 s in controls. Biochemical fractionation showed a shift toward G-actin without a change in total actin. Nuclear MRTF-A fell markedly, SRF reporter activity was reduced to about half of the control, and several MRTF-SRF target transcripts decreased. Stabilizing F-actin with jasplakinolide restored MRTF-A localization. These linked measurements support a causal connection between the altered actin pool and transcriptional output in fibroblasts rather than a simple expression correlation.

Skeletal muscle from the same targeted allele shows reduced expression of several MRTF-SRF target genes [29]. Cardiac tissue shows the opposite direction, with increased nuclear MRTF and increased expression of SRF-dependent genes [49]. In cardiomyocyte-specific CAP2 knockout mice, pharmacological reduction of the SRF-pathway output lowered selected SRF targets and the heart-failure marker Nppa and extended median survival from 98 to 116 days [49]. This intervention supports functional relevance of elevated SRF signaling in the cardiac phenotype, but it does not identify which upstream actin mechanism produces that signaling state. Cardiomyocyte-specific CAP2 deletion also causes major conduction abnormalities and sudden death [50]. Cortical neurons from a separate CAP2 mutant line show an increased F/G-actin ratio and abnormal F-actin accumulation [51].

These findings demonstrate that the cellular consequence of CAP2 loss is not uniform. The fibroblast and skeletal-muscle observations point toward reduced MRTF-SRF output, whereas heart and neuronal data are consistent with greater filament accumulation or increased MRTF-SRF signaling. The comparison is strongest among fibroblast, skeletal muscle, and cardiac studies because they used the same targeted allele [29,48,49]. The neuronal result comes from a different gene-trap line [51] and should therefore be treated as supportive context rather than an equivalent genetic comparison. Serum stimulation also restores MRTF-A translocation and SRF activity in CAP2-null fibroblasts [48], showing that even within one cell type the effect depends on signaling state.

The biochemical framework offers possible explanations but does not identify the cause of these differences. Monomer availability, filament nucleotide state, CAP abundance, and competing end-binding proteins could all contribute. A prospective comparison should measure these variables under the same protocol in two cell types that show opposite responses to CAP2 loss. Such an experiment would test whether the biochemical variables predict the sign of the cellular phenotype rather than merely accommodate it retrospectively.

Cardiomyocytes provide a particularly useful system. CAP2-null cardiomyocytes show increased actin incorporation at both filament ends [9]. One interpretation attributes the barbed-end effect to altered monomer availability, whereas later biochemical work raises the possibility of direct barbed-end regulation by CAP [22]. These alternatives can be separated experimentally. If the phenotype is caused by monomer availability, clamping the soluble actin pool should reduce the difference. If direct barbed-end CAP activity contributes independently, part of the effect should remain.

### 5.2. Neuronal Studies Reveal Additional Variables

Neuronal studies identify additional complications that should be separated from the barbed-end model. CAP1 and CAP2 can compensate for one another. In differentiating neurons, CAP2 overexpression rescues defects caused by CAP1 deficiency, whereas CAP2 loss alone has much smaller effects on early differentiation [52]. A phenotype caused by manipulation of one paralog may therefore underestimate total CAP function. This is a direct cellular observation, but it has not been tested specifically at filament ends.

CAP2 oligomerization also changes during synaptic plasticity. Disulfide-linked dimerization involving Cys32 has been linked to cofilin binding, cofilin recruitment to spines, and spine remodeling [53]. This provides cellular evidence that CAP oligomeric state is regulated on a physiological timescale, although it has not been shown that the same regulation changes filament-end activity. CAP1 also cooperates with cofilin1 in spine morphology and shows more modest redistribution during experimental synaptic plasticity [54,55]. Neuronal phenotypes are themselves context-dependent. CAP2 inactivation has been associated with increased dendritic complexity and spine density in one cortical model [51], whereas CAP2 downregulation in hippocampal neurons has been associated with reduced arborization and enlarged spines [53]. These studies differ in preparation and perturbation and should not be forced into a single barbed-end mechanism.

Studies differ in the reported location of the cofilin interaction. Biochemical reconstitution places an important interaction at the N-terminal HFD in the presence of actin [14,35]. Neuronal pull-down experiments using free cofilin reported binding to the C-terminal region [51], whereas synaptic studies implicate N-terminal dimerization [53]. These experiments used different molecular contexts and may therefore detect different complexes. Repeating binding assays with the same CAP constructs in the presence and absence of ADP-actin would help distinguish these possibilities.

Current neuronal data consequently support context dependence but do not validate a specific barbed-end occupancy mechanism. A direct test would combine CAP1 and CAP2 abundance or localization with capping protein, twinfilin, and actin-state measurements in the same neuronal preparation. The key criterion is prospective discrimination. If two neuronal contexts with different CAP phenotypes show similar local occupancy and actin state, the proposed variables would not be sufficient to explain the difference.

## 6. Conclusions and Experimental Priorities

CAP is established as an actin regulator of the soluble monomer pool and the filament pointed end, and two reconstitution studies now provide strong evidence for C-CAP activity at the barbed end [21,22]. The barbed-end mechanism remains less complete than the pointed-end mechanism.

The most secure barbed-end conclusion is that C-CAP accelerates depolymerization and that WH2 is important [21,22]. Three issues remain open. Existing constructs do not isolate a direct CARP contribution from scaffold and valency. The two barbed-end studies reach opposite conclusions about formin unbinding and processivity despite the persistence of increased formin processivity with profilin-actin in the Towsif study. Processive behavior is supported for yeast Srv2ΔCARP, whereas whether mammalian CAP shows a comparable residence time or run length remains to be established.

These questions are experimentally tractable. Matched full-length, ΔCARP, and CARP–actin interface mutants can test CARP contribution. Formin behavior should be compared with unlabeled and fluorescently labeled approaches under matched filament-state and surface conditions, without assuming that profilin-actin accounts for the discrepancy. Mammalian CAP residence at barbed ends should be measured with explicit photobleaching controls and treatment of censored events.

Structural and high-speed atomic force microscopy studies narrow the range of plausible models without resolving these questions. Cryo-electron microscopy resolves the HFD-cofilin mechanism at the pointed end and cofilin-mediated uncapping, but does not show C-CAP at the barbed end [24]. Atomic force microscopy shows that actin loading can alter CAP residence and binding preference [23].

Cellular studies should test whether monomer availability, filament nucleotide state, barbed-end occupancy, and local CAP abundance predict phenotypic differences across matched systems and should evaluate tropomyosin decoration separately as a proposed modifier. The strongest design will compare systems with opposite CAP phenotypes while measuring these variables under a common protocol. Such experiments can determine whether, and in which contexts, features of the reconstituted mechanism operate at filament ends in cells.

**Review Methodology:** This is a narrative rather than a systematic review. The literature was identified through iterative keyword and citation searches of PubMed and Scite, supplemented by backward and forward citation tracing. References and quantitative claims central to the mechanistic comparisons were checked against the primary publications. Because no PRISMA-style systematic search was performed, statements concerning the absence or prevalence of findings in the literature should be interpreted accordingly. The use of artificial-intelligence tools is disclosed in the Acknowledgments.

## Figures and Tables

**Table 1:** Direct comparison of the two barbed-end reconstitution studies [21,22]. Direct readouts, experimental conditions, and technical limitations that affect interpretation are listed side by side.

Feature	Alimov, Hoeprich and Padrick, 2023 [21]	Towsif and Shekhar, 2025 [22]
Filament state	Not intentionally aged. The design approximated rapidly turning-over leading-edge networks.	Aged for 15 min to enrich ADP-F-actin.
Barbed-end depolymerization	3.58 ± 0.59 su/s in control and 12.9 ± 2.42 su/s with 1 µM CAP, about fourfold.	11.0 ± 0.8 su/s in control and 36.2 ± 3.0 su/s with 1 µM CAP, about threefold.
Responsible region	C-CAP; N-CAP was inactive at the barbed end.	C-CAP; N-CAP was inactive at the barbed end.
WH2 evidence	WH2 actin-binding mutant abolished activity.	WH2 identified as the principal driver of C-CAP activity.
CARP test	GST-dimerized P1-WH2-P2 did not reproduce full C-CAP barbed-end depolymerization. GST also changes scaffold geometry and valency.	CAPΔCARP retained substantial barbed-end depolymerization activity. The construct retains N-terminal oligomerization and differs from the GST fragment in architecture.
Formin readout	Formin unbinding inferred from filament growth or fluorescence; formin was not fluorescently labeled.	Fluorescently labeled formin showed increased processivity. The label provides an occupancy signal but may alter formin behavior.
Capping protein	Not examined directly.	C-CAP accelerated capping-protein dissociation.
CAP processivity	CAP residence/processivity was not measured in the principal Alimov barbed-end analysis; full-length CAP2 was not purified for that analysis.	Long residence and processivity were measured with yeast Srv2ΔCARP; fluorescent mouse CAP1ΔCARP showed nonspecific coverslip binding.

## Data Availability

No new data were created in this study. All data discussed are available in the cited primary publications.

## Abbreviations

AFM, atomic force microscopy; CAP, cyclase-associated protein; CP, capping protein; C-CAP, C-terminal CAP region; F-actin, filamentous actin; FRAP, fluorescence recovery after photobleaching; G-actin, globular actin; HFD, helical-folded domain; MRTF, myocardin-related transcription factor; N-CAP, N-terminal CAP region; P1/P2, polyproline motifs 1 and 2; SRF, serum response factor; WH2, WASP-homology 2.
